# Striking a Balance: Physical Activity, Screen-Viewing and Homework during the Transition to Secondary School

**DOI:** 10.3390/ijerph16173174

**Published:** 2019-08-30

**Authors:** Lydia G. Emm-Collison, Sarah Lewis, Thomas Reid, Joe Matthews, Simon J. Sebire, Janice L. Thompson, Ruth Salway, Russell Jago

**Affiliations:** 1 Centre for Exercise, Nutrition & Health Sciences, School for Policy Studies, University of Bristol, 8 Priory Road, Bristol BS8 1TZ, UK; 2 School of Sport, Exercise and Rehabilitation Sciences, University of Birmingham, Birmingham B15 2TT, UK

**Keywords:** physical activity, screen-viewing, children, school transition

## Abstract

Physical activity levels decline, and screen-viewing behaviours increase during childhood and adolescence. The transition to secondary school appears to coincide with a sharp decline in physical activity. Parents have the potential to influence their child’s behaviours, yet little is known about their expectations for their child’s physical activity and screen-viewing during this transition. This paper explores parents’ expectations for their children’s physical activity and screen-viewing as they transition from primary to secondary school, and their proposed strategies for managing these behaviours during this time. Forty-two parents of children aged 10–11 years participated in a semi-structured telephone interviews in July 2017 or March 2018. The interview data were analysed via inductive and deductive content analysis to explore parents’ perceptions of physical activity and screen-viewing during the transition, the reasons for their perceptions, and the strategies they intended to implement to help their child balance their behaviours. Most parents expected both physical activity and screen-viewing to increase during this transition. There were several individuals, social and school-level factors influencing these expectations. Overall, parents felt that helping their child balance their activity levels, screen-viewing and homework would be challenging.

## 1. Introduction

Physical activity is associated with improved physical, psychological and cognitive health outcomes in children and youth, including lower adiposity, lower cardiometabolic biomarkers, and improved well-being [1]. In developed countries, moderate-to-vigorous physical activity (MVPA) declines throughout childhood and adolescence [2], and very few 15 year-old’s engage in adequate levels of physical activity [3]. There is also some evidence indicating a marked reduction in physical activity around ages 11–12 [4] which coincides with the transition from primary to secondary school in the UK. It is also noticeable that, while light-intensity physical activity (LPA) continues to decline between the ages of 12–15, some evidence indicates that MVPA is stable during these years [5], suggesting that a large proportion of the decline in MVPA occurs before the age of 12. The decline in girls’ MVPA is greater than for boys, with evidence indicating it could be a much as double that of boys (e.g., the average decline in average MVPA of 46% and 23% for girls and boys, respectively) [4].

Sedentary time increases both in and out of school-time during childhood and adolescence [5]. Accelerometer-estimated sedentary time has been shown to increase over the transition from primary to secondary school by 10–20 min per day [6]. However, sedentary time represents a combination of different behaviours that accelerometers are unable to distinguish [7]. Screen-viewing is a sedentary behaviour of particular interest in the child and adolescent population. Higher levels of screen-viewing have been shown to be associated with the higher body-mass index, poorer psychological health and poorer academic achievement in children [8,9]. Evidence shows that over half (56%) of U.S. adolescents exceed the recommended 2 h of screen-time per day [10], with some evidence showing that screen-viewing patterns established during adolescence track through to adulthood [11]. However, much of the literature has focused on television viewing, which is unlikely to be representative of modern screen-viewing behaviours [12]. Given the negative health consequences of physical inactivity, efforts to understand the factors that are associated with both physical activity and screen-viewing are important for implementing strategies to promote lifelong health-behaviour habits.

The transition from primary to secondary school is a key life event for many children, and is one that involves significant changes to both the social and physical environment [13,14]. Changes in physical activity and sedentary time during the transition from primary to secondary school are likely to be influenced by a multitude of individual and environmental factors. From an environmental perspective, school-related factors, such as length of break times, opportunities for lunchtime physical activity and a higher prevalence of sports facilities may be associated with less sedentary time and more MVPA time during the school day [14]. Additionally, the potential increase in homework in secondary school is a key concern for children [15] and, whilst it may be academically beneficial [16], it could also mean longer periods of sitting time and less free time that can be spent being active. Qualitative work exploring the factors associated with the decline in adolescent girls’ physical activity during the transition to secondary school highlighted several factors on multiple levels, including the use of opportunities to be active, preferences for alternative activities, how supportive the physical environment is for being active, sense of self, and personal priorities [15]. In the same study, the reciprocal relationship between physical activity and sedentary behaviours was evident, with many of the girls expressing their personal preferences for screen-viewing activities (e.g., watching movies, playing computer games) as reasons for not engaging in physical activity [15].

Whilst previous research has separately explored the factors associated with physical activity and sedentary behaviours, such as screen-time and homework, in the transition to secondary school, there has been a limited exploration of how these behaviours might influence each other. Additionally, little is known about parent’s perspectives on this topic, which is a salient gap as parental support is associated with both the adjustment to the secondary school environment [17] and physical activity behaviour in adolescence [18]. With this in mind, the present study sought to explore: (1) Parents’ expectations for their child’s physical activity, screen-viewing and homework as they transition to secondary school; (2) reasons for these expectations; and (3) strategies that parents planned to use to ensure these behaviours are balanced during the transition to secondary school.

## 2. Materials and Methods

The data used in this paper are from the B-Proact1v cohort [19,20,21]. The wider study explored children’s physical activity, sedentary time and screen-viewing and associated factors throughout primary school. Data were collected on three occasions between 2012 and 2017 when children were in year 1 (ages 5–6 years), year 4 (ages 8–9 years), and year 6 (ages 10–11 years). One thousand two hundred and ninety-nine child-parent dyads from 57 schools participated in year 1, and the same 57 schools were invited to participate in subsequent years. In year 4, 1223 child-parent dyads from 47 schools participated, and 1296 children-parent dyads from 50 schools participated in year 6. In each phase of data collection, children and parents wore a waist-mounted ActiGraph wGT3X-BT accelerometer for five days. Accelerometer data were processed using Kinesoft (v3.3.75; Kinesoft, Saskatchewan, Canada), and participants were required to have at least three days of valid data, including one weekend day, to be included in subsequent analyses. A valid day was defined as at least 500 min of data after excluding periods of non-wear time ≥60 min, defined as 60 min of consecutive zero counts, and allowing for up to 2 min of counts between 0–100 [3]. Population-specific cut points for children [22] were used to derive average daily minutes of moderate-to-vigorous-intensity physical activity (MVPA). Children’s height and weight were also measured.

**Figure 1:**
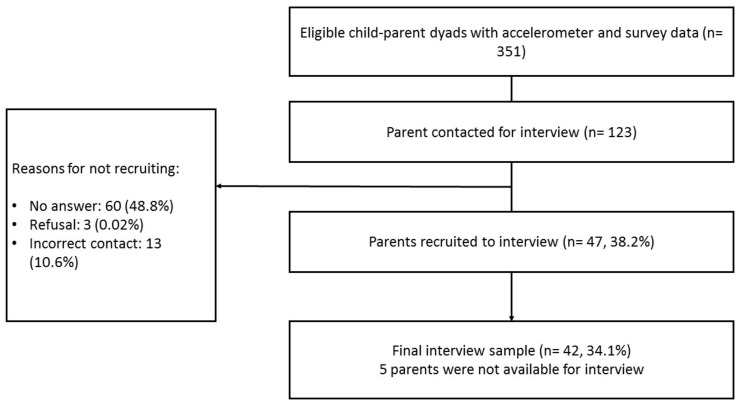
Study flow of participants.

This paper presents qualitative data collected from a sub-sample of these parents in July 2017 (20 parents) and March 2018 (22 parents). Participants were sampled based on the child’s average minutes of MVPA per day. Participants with at least three days of valid accelerometer data were stratified into three groups of low (<51.0 min/day), mid (51.0–71.8 min/day) and high MVPA (>71.8 min/day). These groups were subsequently split by gender to give six groups. To ensure that each group was distinct, only parents of participants with low and high levels of MVPA were eligible for interview (*N* = 351). Within each group, parents were contacted in a randomised order, and recruitment continued until theoretical saturation was met, identified when no new themes emerged in the interviews. In total, 123 parents were contacted, of whom 47 (38.2%) agreed to participate and 42 (34.1%) completed an interview (Figure 1). Semi-structured telephone interviews were chosen as the method due to their cost-effectiveness and the flexibility they offer both participants and researchers [23]. Interviews were conducted at the participant’s convenience, and all participants provided verbal consent at the start of the interview. Interviews were recorded using an encrypted digital voice recorder (Olympus DS-3500). Participants received a £10 gift voucher following the interview as recognition of their time. Ethical approval for the study was granted by the School for Policy Studies Research Ethics Committee.

### 2.1. Interview Data

The semi-structured interview guide was developed and refined by the research team. The interview guide was informed by previous research and gaps in current knowledge. The interview was designed to understand parental expectations for their child’s physical activity and screen-viewing over the transition to secondary school, the factors that they felt would influence patterns in these behaviours, and any strategies they were considering as a way of managing these behaviours. Specific topics that were explored included parents’ expectations for their child’s physical activity and screen-viewing at secondary school, the types of activity and screen-viewing that parents anticipated their child engaging in at secondary school, and parent’s plans for managing physical activity and screen-viewing at secondary school. Example questions included ‘How do you think [child’s] physical activity in secondary school will compare to primary school?’, ‘Do you have any plans for how you might manage [child’s] screen-viewing when they go to secondary school?’, and ‘How do you feel that the school routine and homework schedule at secondary school will differ from primary school?’. To allow participants to shape the direction of the interview, questions were phrased in an open and non-leading manner, and interesting responses were followed up with additional questions. Two male researchers trained in qualitative methods conducted all interviews (JM, TR), and throughout data collection they met regularly with the wider research team (LEC, RJ) to discuss the questions and refine the interview guide. Researchers’ interpretations of participant responses were checked for accuracy during the interviews. At the end of the interview, the researcher’s interpretation of the key findings were summarised, and participants were given the opportunity to comment further on any of the topics discussed.

### 2.2. Demographics

Parents completed a questionnaire, online or on paper, providing parent and child gender and child date of birth. Parents also provided their home postcode, which was used to assign an Index of Multiple Deprivation (IMD) score to each child as an indicator of socio-economic status. IMD scores are based upon the English Indices of Deprivation [24], and higher scores indicate greater deprivation.

### 2.3. Data Analysis

Interviews were transcribed verbatim by a university-approved transcription company. Prior to anonymisation and analysis, all transcripts were checked for accuracy by a member of the research team (LEC). Anonymised transcripts were entered into QSR NVivo 11 (QSR International, Warrington, UK) for analysis. The framework method [25] was used, allowing themes to be developed both from the participants’ experiences (inductive) and from existing literature (deductive), via several stages of analysis: Familiarisation; coding; framework development; framework application; and interpretation. In the familiarisation stage, three researchers (LEC, SL, TR) listened to audio recordings and read all interview transcripts to immerse themselves in the data. The researchers then independently read three transcripts and provided line-by-line annotations to describe key concepts within the data. The annotations were discussed, and with the lead investigator (RJ), the researchers collaboratively developed a coding framework to apply to subsequent transcripts. This process was repeated with three more transcripts, and the framework was refined accordingly. The process continued until no further refinements were required, and the final framework was applied to all the transcripts by the researchers (LEC, SL, TR), and after which the research team met regularly to ensure the framework was consistently applied. Any disagreements in coding were discussed with the wider project team until consensus was achieved. Following coding, hierarchies of themes were created, and summaries with accompanying quotes for each category were extracted to aid reporting. Participant characteristics based on questionnaire and accelerometer data are presented using means and proportions (Table 1). Further, to contextualise the data, the parents’ relationship to the child, child gender and level of MVPA are provided alongside each quote.

## 3. Results

Table 1 outlines the characteristics of the interviewed parents and their children. The average age of the parents was 42.5 years, half of the parents were female, and 95% were white British. Parents of highly active children engaged in more physical activity and had a lower BMI than parents of low active children. Low active children lived in more deprived areas than highly active children.

Most parents believed that the move to secondary school would facilitate their child engaging in more physical activity (*N* = 28) or similar levels (*N* = 8) when compared to primary school. Six parents expected their child’s activity to decrease over the transition to secondary school, but five of these parents felt this change would be temporary whilst their child settled at school. Parents felt similarly regarding screen viewing, with most expecting their child’s screen-viewing to increase (*N* = 20) or stay the same (*N* = 8) in secondary school.

### 3.1. Reasons for Parental Expectations for Physical Activity and Screen-Viewing

Reasons for parents’ expectations of their child’s physical activity and screen-viewing over the transition to secondary school were varied, with several individuals, social, and school-related factors that parents felt would influence these behaviours.

#### 3.1.1. Child Independence

For many parents, active travel to and from school was a key behaviour that they felt would lead to an increase in their child’s physical activity. This was generally due to children engaging a greater amount of walking or cycling to and from school, primarily due to increased child independence, as well as convenience for parents.

“*I’m quite a firm believer when they get to secondary school, that’s where they do need to have their freedom, secondary school I don’t believe um, they need their mums dropping them off outside the school gates … So I think it’s important that she has her freedom and she has the responsibility of getting herself to school and getting herself back safely.*”

(Interview 14, Mother of Low PA girl).

“*I’m not going to be able to take her, so she’s going to have to start taking herself.*”.

(Interview 16, Mother of High PA girl)

However, for some parents, the locations of both home and secondary school meant that their child would be engaging in less active travel.

“*Because it’s so far away he’s gonna get on the school bus. We have spoken about riding his bike but he’s not road safe, so it might be something that he does in the future.*”

(Interview 22, Mother of Low PA boy).

Some parents felt that increases in their child’s independent active travel might also have a positive impact on their child’s screen-viewing, due to spending less time at home.

“*I think [screen-time] will reduce a bit because I don’t think she’ll have as much time because her school day with the longer walk each end her school day is going to be longer, she’s going to be more tired because she’s going to be a lot more, a lot more walking to and from school and a lot more thinking so is she going to be mentally more tired.*”

(Interview 3, Mother of Low PA girl).

However, for most parents, there was a general concern about the increased amount of time their child would be spending alone, either at home or whilst travelling to and from school, with parents anticipating their child would choose to engage in screen-time during this period.

“*It will be the case where he’ll have more [screen-time], he’ll probably have two hours after school in the evening before parents get home and the possibilities are that he’ll use that as screen time.*”

(Interview 41, Father of Low PA boy).

“*So [screen-time] could go up and he will get, if we move close to school he will get a greater level of independence so there’ll be more time when the two children come home alone and they’re just at home and sort themselves out. So, yeah, I guess [screen-time] probably could increase*”.

(Interview 1, Father of High PA boy).

Some parents felt their child was already developing more independence and, as a result, were finding it increasingly difficult to control the amount of time their child spends screen-viewing, and were surprised at how technology-oriented children of this age already are.

“*Because, that’s what the children of today are all about, you know it’s all about technology and communication, between you know what, I was at parents’ evening the other night and I overheard her school friends, her classroom friends talking about Twitter and asking if each other are on Twitter and Facebook and I was quite surprised at you know what they were saying.*”

(Interview 33, Father of Low PA girl).

#### 3.1.2. Social Factors

There were several social factors that parents felt would impact their child’s physical activity and screen-viewing at secondary school. Many parents felt the transition to secondary school itself, specifically the initial period of social adjustment, would reduce their child’s opportunity and willingness to engage in physical activity:

“*I think initially possibly less while he finds his, finds his friendship groups and, and confidence in a new school, to kind of join in group activities but I think, I think once, once we’re over that, he’ll probably, he’ll probably do the same sort of thing, you know?*”

(Interview 23, Father of High PA boy).

“*The only thing I can think of is that, there’s more of a social side of things isn’t there, you’re trying to fit in with everything and be social, so… club after school or done something in school, you might think well, if my mates aren’t doing it, I’ll stay with them, do you know what I mean.*”

(Interview 13, Father of High PA girl).

Some parents attributed this decline to potential peer pressure and, particularly for girls, the desire to fit in with new peers.

“*At secondary it’s completely switched around, you’re the youngest, erm, there’s that whole social thing about not standing out too much from the crowd so if you’re, if you’re you know, just walking round chatting to people you’re not standing out, you’re not going to be teased for playing babyish games, all that sort of thing and I think for [child] that’s quite, she’s quite acutely aware of all of those sorts of things.*”

(Interview 12, Father of Low PA girl).

“*I think it does have a lot to do with it, yeah, because my eldest was quite sporty before she went to secondary school and she was quite tomboy-y and then she then started to wear the make-up and straighten the hair and she didn’t want to ride her bike or she didn’t want to run anywhere and like the kick boxing sort of, dwindled out and, it wasn’t cool to be sporty.*”.

(Interview 16, Mother of High PA girl)

Parents felt that peer pressure would have a specific negative influence on girls’ physical activity, due to a gendered societal view of physical activity as a male behaviour.

“*I think one of the biggest reasons is how they look to their peers, like girls are supposed to be like, all girly and not be sporty or…like I don’t know, not competitive, in the sports world they’re more competitive in like the hair and make-up world…*”

(Interview 16, Mother of High PA girl).

“*I think boys are much more likely to … to be involved in stuff where they’re kinda testing out all sorts of things aren’t they, about their identity and their um, you know, physical prowess, all of those things, and that kind of pre-puberty and puberty really and girls aren’t encouraged to do that. Well it’s a real—you know, it’s something I’m really-really conscious of because of my older daughter, and I just think it’s a—it’s a real shame that, you know, girls are put off sport at that sort of age, at that sort of 11, 12, 13 and stopped getting involved in things and, you know, it’s – yeah, it’s part of—it seems to be cultural doesn’t it.*”

(Interview 26, Mother of High PA girl).

Parents also felt that increased peer pressure at secondary school might lead to increases in screen-viewing. This was generally due to pressure for their child to have their own mobile phone, the introduction of social media, as well as parents not wanting their children to miss out on opportunities to build friendships.

“*At the moment he hasn’t got a mobile phone you know again I think it will be peer pressure there, we will have to give in and get him a mobile phone, I don’t think he needs one at the moment but yeah with secondary school I think unfortunately it just gets you know younger and younger they need these things so yeah so that will increase as well.*”

(Interview 18, Mother of Low PA boy).

“*A lot of that may be if some of her friends or new friends are involved in the Snapchat or the Instagram or a certain app or—and they’re talking about it during the day or even a game that they’re playing and they’re talking about it during the day and she’s involved in that, it could—I would say probably she will come back and start doing those things so that she can join in that conversation, I think that’s a natural thing. So yeah, that could possibly increase it um, depending on what, yeah, the others do in high school.*”

(Interview 14, Mother of Low PA girl).

#### 3.1.3. School

The majority of parents attributed the expected changes in their child’s physical activity levels to the difference between the primary and secondary school environment. This was particularly evident from parents who expected their child’s physical activity to increase during the transition. Many parents anticipated that secondary school would afford their child more opportunities to participate in a wider range of organised activities, due to the facilities available in a larger-site school.

“*In the secondary school he’ll have far more opportunities. It’s a much bigger school ‘cause the one we are at the moment is an extremely small school. We’ve got about a hundred kids at his school, to over a thousand, or about a thousand. So there’s a lot more opportunities, that’s why he chose the school because it’s got Astroturf pitches.*”

(Interview 15, Father of Low PA boy).

“*He’ll have the opportunity to join the rugby team at secondary school yeah I think it will be a lot more varied amount of sports activities there so you know there’s an opportunity for him to actually get an interest in other sports as well.*”

(Interview 18, Mother of Low PA boy).

However, not all parents expected the transition to offer a wider variety of opportunities for their child, and one parent expressed particular concern regarding the availability of after-school activities for their child.

“*In terms of the after-school clubs I am concerned it’s going to go down…because most football trainings that he does, I pay for, finish at age 11…So unless there’s school activities he can do at the senior school on an evening then his level of activity is gonna go down.*”

(Interview 34, Mother of High PA boy).

Aside from participation in organised activities, several parents felt their child’s physical activity would increase due to incidental behaviour associated with being at a larger school.

“*Well again there’s a lot more of like, walking around the school as well, because obviously they’ve got to walk from class to class, so they’re not going to be stuck in the one class.*”

(Interview 16, Mother of High PA girl).

“*The whole set up of secondary school, you are going to be moving around more, lesson change, etc., it’s a bigger campus, [yeah] you know so she’s going to be, everything’s on a larger scale isn’t it, which therefore involves more moving around.*”

(Interview 12, Father of Low PA girl).

One of the important school-related concerns parents had, regarding their child’s physical activity and screen-viewing behaviours at secondary school, was the expected increase in homework. However, for most parents, the way in which homework might influence physical activity was uncertain, with parents feeling it could either inhibit or facilitate their child engaging in more activity.

“*I don’t know. It can go either of two ways really. It might overwhelm him and he’s just too tired with it all, so that he does just wanna sit down and not do anything, or it might push him to be more active.*”

(Interview 22, Mother of Low PA boy).

“*I suppose it could do, bearing in mind obviously she’s got to do, sort of, sit inside and do homework every day hasn’t she, do you know what I mean in the evening, so, but hopefully, like I said, if this homework only takes half an hour, an hour, she should still be able to go out and do stuff.*”

(Interview 13, Father of High PA girl).

Some parents felt that homework would not influence their child’s physical activity, due to participation in organised activities that would not be compromised, and rather felt that time spent completing homework was more likely to replace screen-time.

“*He will still be doing his clubs, or that might be at least twice a week, we’ll still expect him to be doing some physical sort of club. At the end of the day, if he’s got extra homework, it’s the screen time that gets eaten into, not his physical activity.*”

(Interview 10, Mother of High PA boy).

Yet many parents felt the increase in homework would increase their child’s screen-time. This was specifically due to expecting a lot of homework to be completed on the computer, although parents did distinguish between education and leisure-time screen-viewing.

“*It depends because obviously now, at secondary school, I would say there’s gonna be more on the laptop which is what I’ve seen an increase for my son, a lot more research which he does on his phone so she will probably do on her laptop … the homework side of it, it could – it could make it increase.*”

(Interview 14, Mother of Low PA girl).

Parents spoke of some differences in how they felt the increase in homework might influence boys’ and girls’ physical activity differently. This was due to perceptions of boys’ preferences for being outside and active rather than indoors doing school-work.

“*All I can say is that boys are probably just gonna want to be out and play ‘cause that’s what they do at that age, and the girls probably will just get on with it and do their homework. That’s my opinion and I know it’s very sexist but…*”

(Interview 22, Mother of Low PA boy).

“*Girls are better than [at] doing things than boys, like homework, so they come home and think, ‘Oh, I’ll get this done.*”

(Interview 30, Mother of High PA boy).

Despite some parents feeling there were gender differences in how homework might affect other behaviours, many of them felt that how their child would cope with the increased workload is down to their own character and parental support rather than their gender.

“*Well they’re all going to get the same amount of homework I suppose. But I suppose it depends, I don’t think you can say it’s all the same for all girls and all boys. It depends on the individual I would imagine on how active that individual is and what they do.*”

(Interview 28, Mother of Low PA boy).

“*I don’t know, I guess that’s very hard to gauge. It would almost be down to how that’s monitored and controlled by parents rather than being able to say yeah it’s different for girls than boys. I mean it’s the aptitude of the individual or their willingness to apply themselves.*”

(Interview 9, Father of High PA boy).

### 3.2. “Striking a Balance” between Physical Activity, Screen-Viewing and Homework

Despite most parents expecting their child’s physical activity to increase, it was clear that parents felt there was the potential for the increased workload at secondary school to influence physical activity and screen-viewing. Parents expressed the need to find a balance between these activities, and they identified that they need to play a key role in helping their child to balance their school work and other activities, with some recognising the potential benefits of maintaining healthy levels of physical activity on homework and productivity.

“*I think that…the extra demand of homework can actually stop children being able to spend that time playing, going out and doing activities. They spend that much time doing homework they haven’t got time to go out, go and visit their friends and going out for a walk so, it can happen but personally I don’t want to see that happen when it’s a question of trying to strike a balance… I want my child to find the thing that she’s good at and work hard on them and not worry about things she’s not so good at so that she’s got time in her life to do other activities.*”

(Interview 27, Father of High PA girl).

“*It’s obviously necessary that she’s going to have to sit still to do some studying which at the moment she doesn’t really, so yeah, like I say, I think I’d try and kind of make up for that by doing bike rides at the weekend or maybe trying find her another you know dance class that she fancies doing or take her to trampolining places.*”

(Interview 25, Mother of High PA girl).

With the expected changes in behaviours during the transition to secondary school, several parents spoke of strategies they would adopt to try and help them achieve this balance. Many of the strategies revolved around parents monitoring and controlling the amount of time their child spends screen-viewing, suggesting alternative activities to their child and removal of devices if school-work is not completed.

“*Encouraging him to do different things, I suppose again sort of anything that’s not sort of watching telly or on his Xbox I suppose. Encourage him to be with friends and different activities.*”

(Interview 28, Mother of Low PA boy).

“*Well we’ll still apply the same restrictions during the school day so no Xbox or anything like, reduce his gadgets, he’s done the homework and the good thing with the school is that they send emails and tell us if there’s interventions or detention or anything about the children there. So, you get a good idea of what their homework is and their learning ability. As soon as you get anything negative coming back then you can apply restrictions. And I’ve got no hesitation of taking away the Xbox controls or the power cable.*”

(Interview 39, Father of Low PA boy).

Some parents identified that physical activity could be used to help their child get their homework completed more efficiently, and therefore, felt it was important that physical activity was encouraged alongside school-work.

“*But the Secondary school, it’s one of those things, they will have to spend more time doing homework…But again, sort of try and engage in getting the homework done, you know, let’s go out and get some fresh air, or let’s go out and sort of do something and blow away the cobwebs. It’s quite difficult because sometimes the piece of homework can take one child sort of 20 min and it can take another child 40 min.*”

(Interview 8, Mother of High PA girl).

Outside of parents encouraging their child to engage in alternative activities to ensure behaviours are balanced, several parental strategies centred more on their child continuing to develop independence, with the implementation of techniques to facilitate better time management and prioritisation of activities.

“*No, I guess we’ll see how much [homework] she’ll have and then we will just talk it through with her really about the best way to do it and try and get her to understand its best to sort of keep on top of it than letting it build up, something like that.*”

(Interview 42, Father of Low PA girl).

Similarly, it was evident that, for many parents, homework would be the priority, and ensuring that their child completed any school-work prior to engaging in other activities (particularly screen-viewing) as central to achieving balance.

“*I’m not too sure how much it entails, what the school will expect and at the end of the day [homework] takes priority over any gaming.*”.

(Interview 10, Mother of High PA boy)

“*I think he’s, obviously it’s going to be a big jump from what he gets homework-wise now to what he’s going to get. I think it’s going to have to be sort of managed to say, ‘Right, you do your homework first and then you get your screen time’*”

(Interview 28, Mother of Low PA boy).

“*Yeah, ‘cause there’s more homework at secondary school and if she gets into the habit of doing the homework near enough straight away then that sort of frees her up rather than leaving it to the last weekend or the last moment so it’s all one panic.*”

(Interview 5, Father of High PA girl).

Conversely, a few parents felt that homework should fit around their child’s physical activity, making use of the journey between clubs or waiting for siblings to complete activities to complete school-work.

“*I suppose it can impact on them. But again, that’s how we try and work around these things and obviously try and do homework at quiet times, if as I say we’re kinda multitasking, where we’re dropping one of at a sport or a club we would generally do other things or catch things out. You know, we take [him] to rugby, his sister does some reading or some drawing while we’re waiting for him at rugby.*”

(Interview 15, Father of Low PA boy).

## 4. Discussion

Most parents expected both their child’s PA and screen-viewing to increase during the transition to secondary school. Whilst parents’ expectations for their child’s screen-viewing are consistent with existing evidence [5,6], their expectations for physical activity do not align with evidence that has consistently shown a steady declining trajectory in children’s activity levels [2,4]. Parents identified a variety of individual, social and school-level factors that might influence their child’s activity and screen-time during the period of transition, highlighting the complexity of these behaviours. An overview of the main findings and their implications are presented in Table 2.

Parents’ expectations that their child’s physical activity would increase during the transition to secondary school was commonly attributed to an increase in active travel to and from school. This is consistent with evidence indicating that active travel does contribute to children’s overall daily MVPA [27]. However, the location of secondary school and, more specifically, the distance between school and home and perceptions of road safety, could prevent active travel. Distance and safety perceptions have been shown to influence whether children are allowed to walk to school in several studies [28,29,30], suggesting that there is a need for changes to neighbourhood infrastructures to improve safety for children. Targeting environmental features, such as road speed, presence of pedestrian crossings and cycle lanes may enable more children to engage in active travel, and thus, increase daily physical activity at the population level [31,32]. Additionally, for some children, road safety and cycling training may be beneficial. The increased independent mobility that accompanies children’s ability to walk to and from school has also been shown to be associated with the time they spend playing outdoors [33]. Therefore, encouraging children to engage in active travel may have additional benefits for overall physical activity levels beyond the specific contribution of walking or cycling to and from school. Regardless of the mode of transport to and from school, parents largely acknowledged that their child would have greater independence at secondary school. For some parents, this led to concerns regarding their child’s level of screen-viewing, as their increased independence would mean longer periods of being home alone, which parents anticipate being used for screen-time. Evidence suggests that children who have limited access to local neighbourhood facilities (such as parks and youth clubs) engage in more home-based sedentary behaviour [34]. With this in mind, there is a need for more evidence-based and community-led strategies to increase physical activity and reduce sedentary time in rural populations.

Many parents felt their child’s physical activity would be inhibited temporarily during the transition to secondary school. One reason for this expectation was the social adjustment that is inherent to moving schools. The transition from primary to secondary school entails significant changes to the social environment [13,14], and thus, represents an important life event for all children. Whilst many children look forward to the prospect of developing new friendships, qualitative work highlights that they also have concerns regarding how these peer-relationships will develop [35,36]. Evidence indicates that children cluster in friendship groups with similar physical activity levels [37,38], indicating that friendships are associated with physical activity. Therefore, whilst parents were confident that as friendship groups were established their child’s physical activity levels would return to previous levels, this may not be the case for all children and may depend on the relationships they build in the early weeks of secondary school. Previous qualitative studies have shown that friends provide support for physical activity by co-participating in activities, modelling being active, and providing verbal support, with these factors being particularly integral for the initiation of new activities and joining of new clubs [39]. Interventions using peer-relationships to increase physical activity engagement have been shown to be beneficial, particularly for girls [40,41]. This indicates that friendships may be particularly important to physical activity levels during this transition, when new opportunities are presented to the children. Therefore, strategies that help children to build friendships during the transition period and encourage positive experiences of physical activity with friends may be effective for increasing youth physical activity engagement.

Parents of girls were more likely to expect their daughter’s physical activity levels to decline, due to fears around peer pressure and a desire to integrate with their peers. These fears were specific to girls, due to perceptions of what is considered ‘feminine’. Gender norms surrounding girls’ physical activity are complex, with many girls reporting that they enjoy being physically active, understand the benefits of an active lifestyle, but simultaneously feel great pressure to appear ‘feminine’, which is not seen to be conducive with being active [42]. It has been suggested that focusing on fun rather than athletic ability might help to engage more girls in a range of physical activities [43]. Parents were also concerned about how peer-pressure might influence their child’s screen-viewing, with expectations that their child would want to engage in more screen-time, specifically gaming and social media, due to wanting to fit in with their peers. Children have greater access to a range of screen-viewing devices, which has led to an increase in the time they spend screen-viewing [44,45,46]. Most evidence indicates that screen-viewing can be harmful to children’s development and well-being [47,48], but much of this research has been focused on traditional forms of media, such as television viewing. Whilst the literature concerning interactive forms of media (e.g., multi-player video games and social media) is in its infancy, initial findings suggest there may be both positive and negative consequences related to these behaviours [49]. For example, there are consistent associations between social media use and the presence of mobile devices in the bedroom and sleep disturbances in older children and adolescents [50]. However, there is also emerging evidence indicating that interactive digital media, and particularly social media platforms, can be used to facilitate social development and improve mood [51], and may, therefore, be a useful tool to improve physical activity engagement; but more research is required to ascertain the long-term effects of social media use.

Parents felt the new secondary school environment would have a positive impact on their child’s physical activity, due to the enhanced facilities and a wider range of activity options. Whilst there is evidence showing that more opportunities for physical activity engagement, such as through extra-curricular activities and inter-mural sports are associated with greater physical activity engagement in children [52], the literature exploring associations between school facilities and child physical activity in secondary schools is inconclusive, with some evidence that school facilities are associated with physical activity in boys only [53]. Therefore, the influence parents expect the change in the school environment to have on their child’s activity may be unwarranted. Where most school-related changes during the transition were viewed as positive for children’s physical activity, parents were concerned about the impact that the increased workload would have on both physical activity and screen-viewing. Parents had mixed views regarding how homework would influence their child’s activity levels, but previous qualitative and quantitative evidence has found that homework is a barrier to being physically active for children transitioning to secondary school and throughout adolescence [15,54,55]. For parents who felt the increased homework would not impact their child’s physical activity, their main reason was due to the child’s participation in organised activities that would remain part of their day. There is some evidence to suggest that participation in organised activities in childhood is associated with higher levels of physical activity in adulthood [56], indicating that structured activity patterns may persist over time and so may not be altered by additional pressures. Therefore, encouraging more children to participate in organised physical activities may promote lifelong behavioural habits. Parents consistently felt that the increase in homework would mean an increase in their child’s screen-viewing. Technological developments result in an increasing proportion of homework being completed on a computer or tablet, thus, inherently increasing screen-time, with evidence indicating that during the transition to secondary school, homework time and computer use both increases [57,58]. Parents did distinguish between recreational and educational screen-viewing, but further research is required to ascertain the relative effects these types of behaviour on health outcomes.

All parents expressed an intention to monitor their child’s physical activity, screen-viewing and homework during the transition to secondary school as an attempt to ensure that these behaviours were well-balanced. However, many of the parents felt that achieving balance was going to be a challenge and expected that balancing their own desire to support their child’s independence with their concern about their child’s preferences (e.g., screen-viewing over physical activity and homework) would be particularly difficult. Many of the parents reported having already implemented practical strategies to try and achieve a balance, including suggesting alternative activities to screen-viewing (physical activity and social activities) and removing screen-viewing devices. Logistical support and parental modelling of physical activity at ages 11–12 have been shown to be associated with lower levels of screen-time and higher levels of physical activity at ages 13–14 [58], so the practical strategies that the parents suggest may be effective for balancing behaviours, provided the parents also balance their own behaviours. In addition to practical solutions, several parents spoke of beginning to develop their child’s independence with regards to physical activity and screen-viewing throughout the final year of primary school. Strategies that encourage children to self-monitor and regulate their behaviours may be most effective, with evidence indicating that self-regulation of screen-viewing is associated with less social-media use [59]. Additionally, encouraging children to manage their own transition from primary to secondary school has been cited as an important strategy for reducing transition-related anxiety [60]; therefore, supporting children to identify their own needs in terms of balancing behaviours may reduce the impact that the transition to secondary school has on their physical activity and screen-viewing.

### Strengths and Limitations

This study has several strengths, including the recruitment of a diverse range of parents from a variety of socio-economic backgrounds and with children whose physical activity levels range from low to high. Notably, the sample had equal representation of mothers and fathers, despite it being difficult to recruit fathers into research projects [61]. However, the study has several limitations, including the limited ethnic diversity within the sample, which inhibits the generalisability of the findings to families with different ethnic and cultural backgrounds. A notable limitation is the studies reliance on parents’ prospective expectations for their child’s physical activity and screen-viewing as they progress to secondary school. A longitudinal qualitative design, gaining perspectives before and after the transition to secondary school, would provide greater insight into how expectations match the reality of this transition, and could provide useful information to inform future strategies to support children and parents during this time.

## 5. Conclusions

The present study highlights several individuals, social and school-based factors that parents feel will influence their child’s physical activity and screen-viewing during the transition to secondary school. These findings indicate a need for more community-based opportunities for secondary school children to be active after school, particularly in rural areas and strategies to help children establish relationships with secondary school peers as early as possible. All parents held the expectation that balancing physical activity, screen-viewing and homework during the transition was going to be challenging, and therefore, there is also a need for more family-based support during this time to ensure that healthy behavioural patterns are established.

## Figures and Tables

**Table 1:** Characteristics of the sample of parents (*N* = 42) and their children by the group and overall.

	High MVPA Boy (*N* = 10)	Low MVPA Boy (*N* = 11)	High MVPA Girl (*N* = 10)	Low MVPA Girl (*N* = 11)	Overall (*N* = 42)
	Mean (SD)/%	Mean (SD)/%	Mean (SD)/%	Mean (SD)/%	Mean (SD)/%
**Child characteristics**					
Gender (% female)	-	-	-	-	50.0%
Age (years)	10.7 (0.5)	10.8 (0.4)	10.9 (0.3)	10.8 (0.3)	10.8 (0.4)
BMI z-score ^1^	0.10 (0.6)	0.39 (1.4)	0.44 (0.7)	0.87 (0.9)	0.45 (1.0)
MVPA (mins/day) ^2^	89.1 (13.7)	46.3 (6.2)	83.3 (14.2)	40.2 (12.5)	64.6 (25.0)
Sedentary time (mins/day)	427.3 (68.6)	472.5 (42.1)	466.2 (69.4)	471.1 (51.0)	459.2 (59.9)
**Parent characteristics**					
Gender (% female)	50.0%	36.4%	70.0%	55.0%	50.0%
Age (years)	44.0 (6.3)	42.6 (5.8)	44.1 (6.8)	39.6 (4.0)	42.5 (5.9)
BMI (kg/m^2^)	24.5 (3.0)	28.3 (7.4)	23.1 (2.7)	25.2 (4.2)	25.3 (4.9)
IMD ^3^	11.9 (5.6)	18.3 (18.1)	15.0 (11.0)	22.6 (18.6)	16.9 (14.5)
MVPA (mins/day) ^4^	66.9 (28.2)	53.2 (26.1)	70.3 (20.1)	46.9 (19.4)	58.9 (24.8)
Sedentary time (mins/day)	539.5 (44.6)	525.4 (47.4)	534.8 (82.9)	524.8 (61.8)	530.8 (58.9)
Ethnicity (%White British)	100.0%	100.0%	90.0%	90.9%	95.2%

^1^ Age- and sex- specific body mass index standard deviation score [26], ^2^ Child moderate to vigorous physical activity defined as ≥2296 counts per minute [22], ^3^ Indices of multiple deprivation, based upon the English Indices of Deprivation [24], ^4^ Parent moderate to vigorous physical activity defined as ≥2020 counts per minute [3].

**Table 2:** Findings and recommendations for supporting children’s physical activity during the transition to secondary school.

Finding	Recommendations
An increase in child independence and independent mobility during the transition to secondary school may lead to increases in physical activity, but also screen-time	Strategies that offer physical activity opportunities during after-school hours are needed (e.g., after school clubs, active travel) both in primary and secondary school, particularly for children who live in neighbourhoods with limited access to facilities.
The period of significant social adjustment as children transition to secondary school could inhibit their physical activity.	Additional support for children before and during the transition period to enable them to establish relationships with peers as early as possible and provision of opportunities for friends to be active together.
Incongruence between the primary and secondary school physical activity environments may contribute to the decline in physical activity during this time	Greater communication between primary and secondary schools, and between schools, parents and children. Whole-school approaches to physical activity at both primary and secondary schools to set expectations for children and parents during the transition. Opportunities for children to familiarise themselves with the secondary school environment, such as through taster days or holiday clubs in secondary school settings.
Limited parental awareness of strategies to help their child balance physical activity, screen-viewing and homework	Working with parents prior to the transition to set realistic expectations and establish plausible and implementable strategies to help their child cope with the increased workload.
